# Swept-Source Optical Coherence Tomography Biometer as Screening Strategy for Macular Disease in Patients Scheduled for Cataract Surgery

**DOI:** 10.1038/s41598-019-46243-3

**Published:** 2019-07-09

**Authors:** Daniele Tognetto, Marco R. Pastore, Chiara De Giacinto, Riccardo Merli, Marco Franzon, Rossella D’Aloisio, Lorenzo Belfanti, Rosa Giglio, Gabriella Cirigliano

**Affiliations:** 1 Eye Clinic, Department of Medical Surgical Sciences and Health, University of Trieste, Trieste, 34129 Italy; 2 Department of Mathematics and Computer Science, University of Trieste, Trieste, 34129 Italy

**Keywords:** Optical imaging, Imaging techniques

## Abstract

The aim of this study was to assess the central macular imaging captured with an optical biometer based on full-eye-length Swept-Source OCT (SS-OCT) scan as a screening strategy for identifying macular diseases in patients scheduled for cataract surgery. 1,114 eyes of 749 consecutive patients underwent a biometrical examination with IOLMaster 700 SS-OCT technology (Carl Zeiss) and conventional Spectral-Domain OCT (SD-OCT) (Spectralis OCT, Heidelberg) device analysis on the same day. Seven examiners graded the scans individually in a full-masked mode. Twenty-five eyes were excluded for media opacities. Among the 1,089 included eyes, statistical analysis revealed a mean Kendall’s Coefficient of 0.83 (range 0.76–0.89). A logistic regression model demonstrated a highly significant correlation (p < 0.001) between the coefficient of concordance and SD-OCT imaging. Intraobserver reproducibility was 0.89 (range 0.86–0.91). Optical biometer SS-OCT scans showed a mean sensitivity of 0.81 and a mean specificity of 0.84. The positive and negative predictive value detected was 0.78 and 0.86, respectively. In order to predict the risk of reduced visual recovery, especially in cases of retinal pathology, optical biometer with SS-OCT scan has proven to be a useful modality for detecting macular structural abnormalities in patients undergoing cataract surgery. Conventional SD-OCT remains mandatory to confirm the presumed diagnosis.

## Introduction

Cataract surgery is the most frequently performed surgical procedure in developed countries^1,2^, providing significant long-term and cost-effective improvements in the quality of life of patients^3^. In the last decades, advances in cataract surgery techniques and new technological developments have led to improved patient safety and satisfaction, resulting in high expectation regarding the refractive outcomes^4^.

According to the accuracy of biometry measurements, the intraocular lens (IOL) power calculation is a crucial step to achieve the targeted refraction. In the last years, a switch from ultrasound-based to optical biometry has been made to provide highly reproducible results. A new optical biometer (IOL Master 700 - Carl Zeiss Meditec AG, Jena, Germany) uses a swept-source optical coherence tomography (SS-OCT) technology to determine the anterior segment biometrical data of the eye. It performs a small central macular scan as well, introduced as quality control of the patient’s fixation during the examination, which may significantly affect the final IOL power calculation.

The macular diseases may also influence the axial length measurement and the final refractive result. Cataract and retinal diseases may progress concurrently in the aging population, and one common challenge before cataract surgery is to detect and report macular disease^5^. Due to the presence of cataract, minor pathologic retinal changes could be missed during preoperative slit lamp fundus examination. Spectral-Domain OCT (SD-OCT) scan offers a noninvasive detailed morphological analysis of the macular structure, which is usually not feasible routinely, especially in high volumes cataract surgery. In this study, the central macular imaging captured with the optical biometer based on full-eye-length SS-OCT was tested as a screening strategy for identifying macular diseases in patients scheduled for cataract surgery.

## Results

1,114 eyes of 749 consecutive patients scheduled for cataract surgery were enrolled in the study. In 25 eyes of 17 patients, the opacities of the cornea or hypermature cataract precluded the SD-OCT acquisition and were excluded. The age of the 732 patients with successful SD-OCT scan performed ranged between 33 and 87 years, with a mean age of 64.3 ± 9.1 (SD) years old.

### SD-OCT analysis

Overall 1,089 included eyes, SD-OCT examination revealed a macular pathology in 449 eyes (41.2%), and a normal macular scan was observed in 640 eyes (58.8%). The detected foveal alterations included 163 epiretinal membranes (ERM, 15.0%), 16 full-thickness macular holes (FTMH, 1.5%), 11 macular pseudoholes (MPH, 1.0%), 9 lamellar macular holes (LMH, 0.8%), 13 vitreomacular traction (VMT, 1.2%), 72 pigmented epithelium detachments (PED, 6.6%), 41 geographic atrophies (GA, 3.8%), 6 retinal detachments involving the fovea (RD, 0.6%). In 127 scans intraretinal fluid (IRF, 11.7%) was observed, 42 scans showed the presence of subretinal fluid, subretinal hyperreflective exudation or vitelliform material (SRF, 3.9%), and in 91 eyes macular drusen were detected (MD, 8.4%). In 142 (31.6%) pathological scans more than one foveal alteration was found. SD-OCT demonstrated a high intraobserver reproducibility both for healthy and for pathological imaging, with a value of 96.1% (615 of 640 correct) and 90.2% (405 of 449 correct), respectively.

### SS-OCT biometer analysis

A mean of 109 (10%, range 82–137) over 1,089 SS-OCT scans were graded as not clear for the bad quality of imaging. Nevertheless, these scans were evaluated, and the suspected diagnosis was provided, if applicable. Related to media opacities, artifacts due to poor fixation or eye movements during the examination, or elevated axial length, an average value of 146 (13.4%, range 134–168) over 1,089 scans were graded not evaluable and removed from the further investigation.

**Figure 1:**
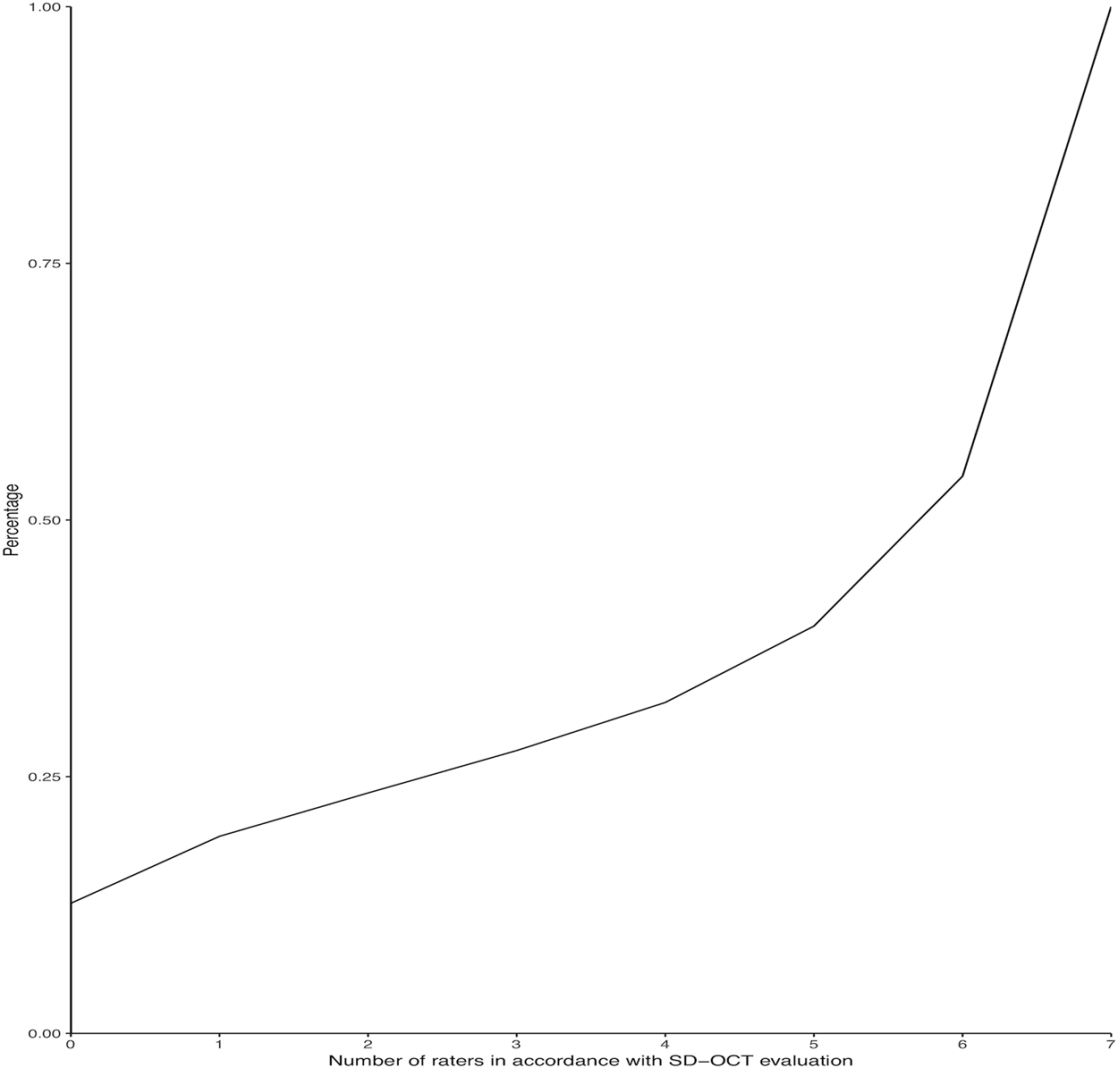
Cumulative curve of agreement between the Swept-Source Optical Coherence Tomography (SS-OCT) biometer readers related to the Spectral-Domain Optical Coherence Tomography (SD-OCT) scans grading. The axis of abscissas shows the number of the raters in accordance with SD-OCT evaluation, from zero to maximum of seven examiners. In the ordinate axis the number of accordance events between the SS-OCT biometer and SD-OCT device scans are reported as percentage.

Statistical analysis of the agreement among the different examiners revealed a mean Kendall’s Coefficient (W) of 0.83 (range 0.76–0.89). A logistic regression model demonstrated a highly significant correlation (*p* < 0.001) between the coefficient of concordance W and the SD-OCT imaging (Fig. 1). For intraobserver reproducibility, high concordance between the baseline and eight weeks apart examination was found, with a mean Kendall’s Coefficient of 0.89 (range 0.86–0.91).

**Figure 2:**
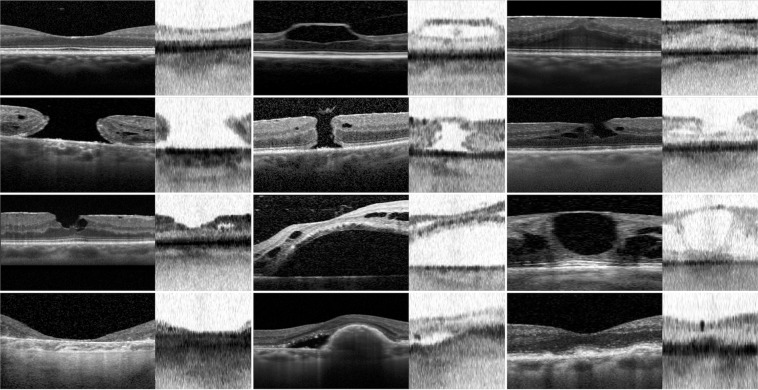
Comparison of the Spectral-Domain Optical Coherence Tomography (SD-OCT) scan in left part of each panel and the Swept-Source Optical Coherence Tomography (SS-OCT) biometry imaging in right part of each panel. (**a**) Healthy eye with normal macular scan. (**b**) Vitreomacular traction and (**c**) epiretinal membrane scans. (**d**) Comparison of SS-OCT biometry and SD-OCT scan for large and (**e**) small full thickness macular holes. (**f**) Lamellar macular hole with intraretinal cysts. (**g**) Macular pseudoholes associated with intraretinal cyst and epiretinal membrane. (**h**) Macula-off retinal detachment at SS-OCT biometry and SD-OCT examination. (**i**) Intraretinal fluid imaging. (**j**) Comparison of SS-OCT biometry and SD-OCT scan for geographic atrophy and (**k**) pigmented epithelium detachment associated with perilesional subretinal fluid. (**l**) Subfoveal drusen greater than 125 µm at SS-OCT biometry and SD-OCT examination.

SS-OCT evaluation between pathological and healthy scans showed a sensitivity of 0.81 (range 0.77–0.83) with a specificity of 0.84 (range 0.77–0.87). Reported accuracy was 0.83 (range 0.79–0.85). The positive and negative predictive value detected was 0.78 (range 0.71–0.82) and 0.86 (range 0.84–0.88), respectively. All biometric SS-OCT evaluation factors for each examiner are summarized in Table 1. A subgroup analysis related to different macular abnormalities was performed (Table 2). Several examples of foveal SD-OCT scans and related SS-OCT biometer imaging are shown in Fig. 2.

**Table 1:** Swept-Source OCT evaluation between pathological and healthy scans for each single examiner.

	Reader 1	Reader 2	Reader 3	Reader 4	Reader 5	Reader 6	Reader 7
True Positive	325	302	323	320	307	325	320
True Negative	479	451	474	478	469	454	424
False Positive	73	101	78	74	83	98	128
False Negative	66	89	68	71	84	66	71
FPR	0.13	0.18	0.14	0.13	0.15	0.18	0.23
FNR	0.17	0.23	0.17	0.18	0.21	0.17	0.18
Sensitivity	0.83	0.77	0.83	0.82	0.79	0.83	0.82
Specificity	0.87	0.82	0.86	0.87	0.85	0.82	0.77
Accuracy	0.85	0.80	0.85	0.85	0.82	0.83	0.79
PPV	0.82	0.75	0.81	0.81	0.79	0.77	0.71
NPV	0.88	0.84	0.87	0.87	0.85	0.87	0.86

Abbreviations: FPR = False Positive Rate; FNR = False Negative Rate; PPV = Positive Predictive Value; NPV = Negative Predictive Value.

**Table 2:** A subgroup analysis related to different macular disease.

Disease	Sensitivity	Specificity	Accuracy	PPV	NPV	PLR	NLR	DOR
ERM	0.69 (0.61–0.76)	0.93 (0.91–0.94)	0.89 (0.86–0.91)	0.66 (0.60–0.72)	0.93 (0.92–0.95)	9.40 (7.17–12.32)	0.34 (0.27–0.42)	27.86 (25.18–29.94)
FTMH	0.94 (0.90–0.99)	0.99 (0.98–0.99)	0.99 (0.98–0.99)	0.75 (0.69–0.84)	1.00 (0.99–1.00)	173.81 (167.86–181.42)	0.06 (0.02–0.10)	2766.00 (2754.81–2773.59)
MPH/LMH	0.70 (0.65–0.77)	0.99 (0.98–0.99)	0.98 (0.96–0.99)	0.61 (0.53–0.76)	0.99 (0.97–0.99)	71.79 (58.27–86.11)	0.30 (0.16–0.39)	236.96 (220.65–256.48)
VMT	0.77 (0.69–0.83)	0.99 (0.98–0.99)	0.99 (0.97–0.99)	0.56 (0.47–0.62)	1.00 (0.99–1.00)	89.42 (0.80–0.83)	0.23 (0.09–0.41)	384.17 (368.19–896.96)
IRF	0.69 (0.59–0.76)	0.78 (0.75–0.81)	0.76 (0.74–0.79)	0.32 (0.29–0.36)	0.94 (0.92–0.95)	3.07 (2.58–3.66)	0.41 (0.32–0.53)	7.58 (7.43–7.70)
SRF/DRm	0.74 (0.58–0.86)	0.78 (0.75–0.80)	0.78 (0.75–0.80)	0.13 (0.11–0.16)	0.98 (0.96–0.99)	3.31 (2.66–4.11)	0.34 (0.20–0.56)	9.81 (9.55–10.14)
PED	0.49 (0.37–0.61)	0.75 (0.72–0.78)	0.73 (0.70–0.76)	0.14 (0.11–0.17)	0.95 (0.93–0.96)	1.93 (1.49–2.25)	0.69 (0.55–0.86)	2.82 (2.67–3.06)
GA	0.44 (0.29–0.60)	0.70 (0.66–0.73)	0.68 (0.65–0.71)	0.06 (0.04–0.09)	0.96 (0.95–0.98)	1.44 (1.13–2.06)	0.81 (0.61–0.96)	1.78 (0.82–2.45)
MD	0.63 (0.52–0.73)	0.75 (0.72–0.78)	0.74 (0.71–0.76)	0.21 (0.18–0.24)	0.95 (0.93–0.96)	2.49 (2.05–3.04)	0.50 (0.38–0.65)	5.00 (4.68–6.31)
MD > *125 µm*	0.68 (0.52–0.81)	0.85 (0.83–0.88)	0.85 (0.82–0.87)	0.19 (0.15–0.23)	0.98 (0.97–0.99)	4.64 (3.59–6.00)	0.37 (0.24–0.58)	12.45 (12.02–12.87)
*MD* < *125 µm*	0.45 (0.30–0.64)	0.70 (0.66–0.73)	0.68 (	0.07 (0.05–0.10)	0.96 (0.95–0.97)	1.47 (1.05–2.12)	0.80 (0.61–0.93)	1.84 (0.91–2.46)

Abbreviations: PPV = Positive Predictive Value; NPV = Negative Predictive Value; PLR = Positive Likelihood Ratio; NLR = Negative Likelihood Ratio; DOR = Diagnostic Odds Ratio; ERM = epiretinal membrane; FTMH = full thickness macular hole; MPH = macular pseudohole; LMH = lamellar macular holes; VMT = vitreomacular traction; IRF = intraretinal fluid; SRF = subretinal fluid; DRm = retinal detachments involving the macula; PED = pigmented epithelium detachments; GA = geographic atrophies; MD = macular drusen with subgroup analysis, related to the drusen size with a cut-off of 125 microns. Data are expressed with the interval estimate (95% confidence interval).

## Discussion

In the past few decades, significant advances in phacoemulsification with IOL implantation have led to improved refractive outcomes and higher patient satisfaction. However, macular changes sheltered by cataract^6^ can reduce surgical success as well as patient and ophthalmologist expectation^7^. Therefore, preoperative assessment is crucial to identify patients with unknown macular disease that may affect final visual outcomes.

An SS-OCT biometer (IOL Master 700, Carl Zeiss Meditec AG, Jena, Germany), allowing two-dimensional imaging of the eye, produce a central 1 mm horizontal retinal scan that may be used for detection of morphologic foveal changes.

In our study, we analyzed the effectiveness of SS-OCT biometer in providing information about the foveal condition to predict the risk of reduced visual recovery after cataract surgery or to obtain the indication for combined phacovitrectomy. Our results demonstrated an overall high sensitivity and specificity of the integrated macular scan to recognize normal and pathological foveal profile, with different predictability according to the distinct macular alterations investigated.

Bertelmann *et al*.^8^ for the first time evaluated the foveal pit morphology during optical biometer measurements using the full-eye-length SS-OCT scan biometer. In this prospective study, 146 eyes were classified into two groups: phakic, non-vitrectomized eyes scheduled for cataract surgery and pseudophakic non-vitrectomized eyes with acrylic lenses. Central retinal thickness (CRT) from IOLMaster 700 was analyzed and compared with standard SD-OCT. The repeatability and accuracy of CRT measurements with the SS-OCT scan biometer discloses acceptable global results related to standard SD-OCT macular scan, but in the phakic subgroup, CRT analysis differed significantly using the two different systems. This difference detected in the group of most significant interest for macular screening could be explained by the lower resolution of retinal B-scans of SS-OCT biometry stressed in case of phakic status or cataract. Besides, another potential influencing factor could be the distortion of the SS-OCT biometry imaging that appears flattered compared with standard SD-OCT foveal scan. Further analysis in Bertelmann *et al*.^8^ study revealed a diagnostic advantage of the IOLMaster 700 in the detection of macular alterations during the preoperative biometrical measurements, especially for ERM, FTMH as well as IRF. Although data on different macular pathologies were reported, none sensitivity and specificity analysis for each alteration have been assessed in that study.

In a consecutive case series of 125 eyes, Hirnschall *et al*.^9^ evaluated the sensitivity and specificity of the IOLMaster 700 for detecting macular disease in eyes scheduled for cataract surgery. Nevertheless, patients with macular pathologies were preferred in order to have at least 50% of the study cohort with pathological foveal alterations. Three examiners graded all biometrical scans and compared to standard macular examination with SD-OCT. The demonstrated interobserver reproducibility between the examiner 1 and 2, 1 and 3, and 2 and 3 was 78.3%, 80.0%, and 86.7%, respectively. A total moderate biometrical scan sensitivity, between 0.42 and 0.68, and a high specificity, ranging between 0.89 and 0.98, was found. During the grading, ERM, FTMH, and IRF were detected in most to all cases. Conversely, GA has been observed not to be easy to identify^9^. Although the study summarized these data, pathology-related subgroup analysis for sensitivity and specificity was not clearly reported.

In our study, we assessed the reliability of the central macular imaging captured with the full-eye-length SS-OCT biometer as a screening strategy for identifying macular diseases in patients scheduled for cataract surgery. The study was designed to reproduce the conditions in which the ophthalmologists work daily in their clinical practice. A high index of agreement between the examiners was found, with a mean W = 0.83. We tested Kendall’s W with a logistic regression model, resulting in a highly significant correlation (*p* < 0.001) between the coefficient of concordance W and the SD-OCT imaging.

In our report, the overall analysis of the biometer in differentiating healthy from pathological scans demonstrated a mean sensitivity of 0.81 and a mean specificity of 0.84. Compared to Hirnschall *et al*.^9^, the lower specificity noticed in our series may be explained by the greater eyes cohort included in the study. A fundamental data observed in our study was the high negative predictive value of 0.86, corresponding to the probability that a patient with a negative test is really a healthy subject. The combined high results related to the sensitivity and the negative predictive value confirmed the IOLMaster 700 as helpful and reliable device to identify healthy eyes with non-pathological foveal structure, providing a predictable and reproducible macular imaging for the normal foveal pit.

The subgroup analysis related to different macular abnormalities revealed high predictability of SS-OCT to detect FTMH, and a moderate-high positive predictive index for pathologies involving the inner retinal layers such as VMT, ERM, and MPH/LMH. Moderate positive predictive values for IRF and SRF/DRm, and low predictability for PED, GA, and MD were found. The DOR evaluation also highlighted similar results. A very high DOR for FTMH, high values for MPH/LMH and VMT, and medium-high values for ERM were found. A moderate index for IRF and SRF/RDm, and low rate for PED, GA, and MD were observed. MD data deserve specific comment because, as reported in Table 2, a further subgroup analysis, related to the drusen size with a cut-off of 125 microns, demonstrated a very different results with a moderate-high index for drusen greater than 125 µm (DOR of 12.45), and low values for lesions equal or lesser than 125 µm (DOR of 1.84).

**Figure 3:**
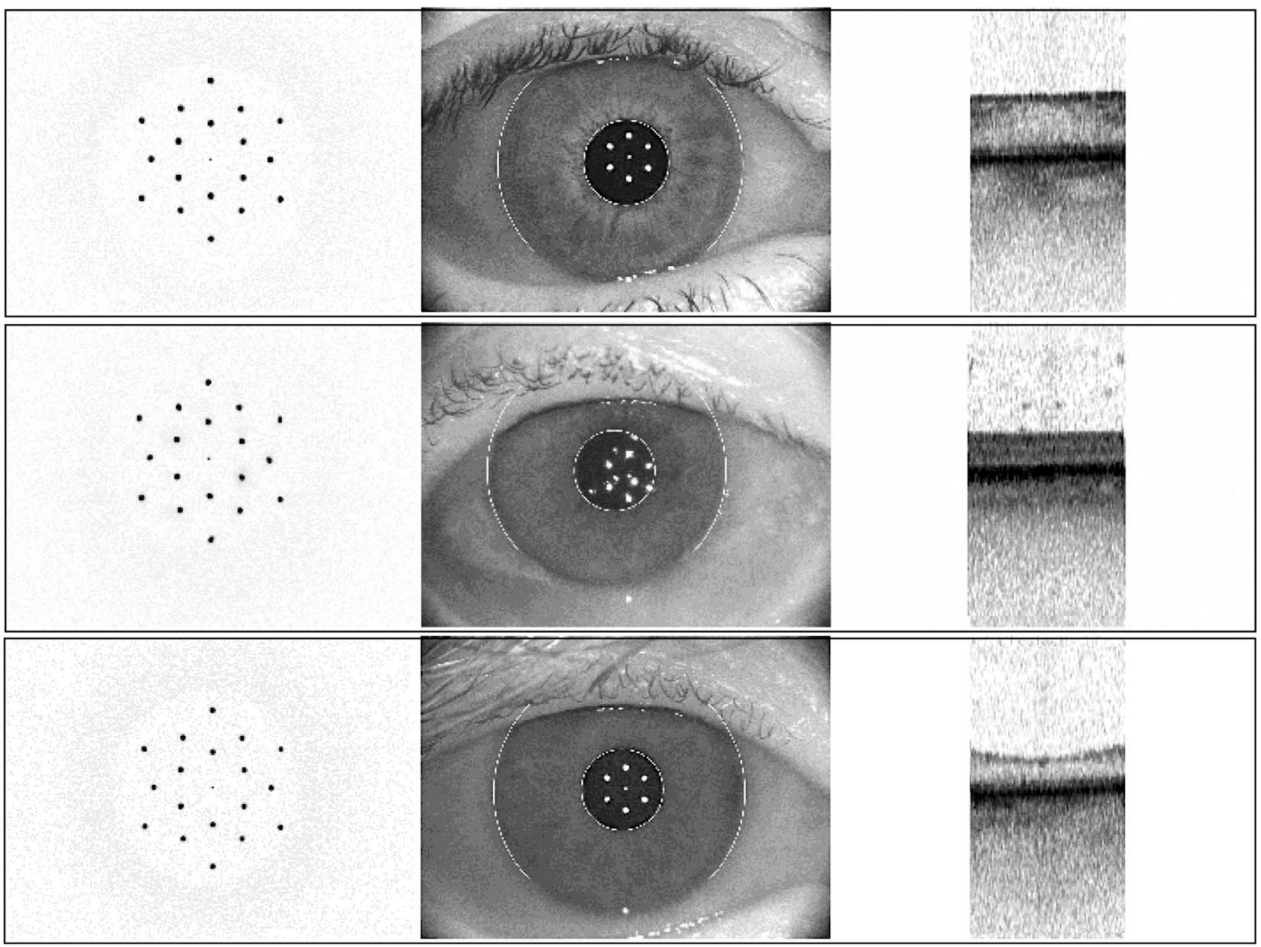
Panoramic external report of the eye at biometer in order to detect the poor fixation condition. (**a**) Good fixation on the panoramic eye view with epiretinal membrane findings at Swept-Source Optical Coherence Tomography (SS-OCT) retinal scan. (**b**) Poor fixation condition at the panoramic anterior segment view related to off-center artefact at SS-OCT retinal scan. (**c**) Good fixation and eye alignment with respect to the pupil center associated with normal macular SS-OCT imaging.

The main limit of the biometer imaging is the small size of the analyzed zone. The central scan zone does not allow to detect any extrafoveal pathologies. In our series, the abnormalities that did not affect the fovea were detected only with SD-OCT examination, and, as expected, none of these cases was reported in the SS-OCT biometer analysis. Another drawback due to small biometric retinal scan is the evidence of poor fixation artifacts, especially for FTMH or GA, pathologies characterized by an extrafoveal point of fixation in the peripheral foveal zone. To avoid misdiagnosis and to clearly recognized this event, the IOLMaster 700 SS-OCT device contains a checking system mainly introduced to reduce the risk of refractive surprises due to incorrect measurements caused by undetected poor fixation. This method is based on a panoramic view of the eye provided by a camera associated with direct visualization of the eye alignment with respect to the pupil center^10^. In our study, the scans not interpretable due to artifacts related to poor fixation or eye movements during the examination were removed from further SS-OCT investigation (Fig. 3). Furthermore, a wider scan width could provide important reference landmarks in the posterior pole^11^ in order to easily identify the off-center scans, without comparing the retinal imaging to the panoramic anterior segment view.

According to the imaging analysis, all examiners enrolled in the study were experienced SD-OCT scans readers, with a daily practice of SD-OCT analysis since at least three years. In their early-stage experience with SS-OCT scans, they reported an immediate and intuitive grading of the foveal imaging resulting in a very fast learning curve.

A normative database as the reference model should be introduced in the biometer report to standardize the foveal pit analysis. This interesting idea should take into consideration the significant changes in the central retinal thickness in normal eye population^12,13^. In addition, we believe an enhancement in the biometer resolution could surely improve the global sensitivity and the specificity of the SS-OCT scans. A quality index to evaluate the status of the OCT imaging added to the normative database could highly increase the screening capability of the device.

In summary, although the SS-OCT foveal scan was developed to analyze the correct alignment of the optical axis during the axial length measurement, the biometer with SS-OCT provided useful information concerning the macular structure. In order to predict the risk of reduced visual recovery and to avoid poor patient satisfaction, especially in cases of retinal pathology, the optical biometer SS-OCT imaging has proven to be an effective, helpful modality for detecting macular abnormalities in patients undergoing cataract surgery with good eye alignment during the examination. Conventional high-definition SD-OCT remains mandatory to confirm the presumed diagnosis obtained with biometer.

## Methods

### Study design

Single-center blinded cross-sectional study performed at Eye Clinic, University of Trieste, Department of Medical Surgical Sciences and Health, between April 3, 2017, and October 27, 2017. All research and measurements were performed according to the Italian bioethical legislation and followed the Declaration of Helsinki and good clinical practice for research involving human subjects. The study protocol was approved by the Institutional Review Board of Azienda Sanitaria Universitaria Integrata di Trieste, and informed consent was obtained from all participants. In the study were included patients scheduled for cataract surgery, who underwent a biometrical examination with IOLMaster 700 (Carl Zeiss Meditec AG, Jena, Germany) and conventional SD-OCT analysis (Spectralis OCT, Heidelberg Engineering, Heidelberg, Germany) on the same day.

The IOL Master 700 (Carl Zeiss Meditec AG, Jena, Germany) is a non-contact optical biometry instrument which combines corneal surface analysis with a 3-zone telecentric keratometer and a full-eye-length OCT scanning for measuring the anterior segment biometrical data, the axial length, and a central 1.0 mm zone macular scan. The length measurements are based on a swept-source frequency domain optical coherence interferometry with a wavelength of 1,055 nm enabling a 44 mm scan depth with 22 µm resolution tissue. The speed of the length measurement system allows acquisition of full-eye-length tomograms at 2,000A-scan per second. Swept source biometry applies optical B-scan technology to determinate the biometrical data. The optical B-scan technology allows cross-sectional visualization of the structure along the visual axis.

### Data analysis

All SS-OCT biometric scans were exported anonymously and randomized by a single external operator (M.F.) to be graded by seven different examiners in a full-masked mode. Three retinal specialists (R.D.A., C.D.G, R.M.), two vitreoretinal surgeons (M.R.P., G.C.) and two experienced residents (L.B., R.G.) individually performed the imaging analysis. To avoid inter-examiner bias, the results were directly assessed. According to the analysis protocol of biometric scans, readers were asked to define if the scan was pathological (response option: yes, no, not clear, not estimated) and what was the suspected diagnosis, if applicable. The three retinal specialists graded all biometric scans eight weeks from the baseline examination to evaluate the intraobserver reproducibility of the SS-OCT imaging.

The SD-OCT scan protocol used was cube 20 × 50 corresponding to a 49-line raster with 120 µm interline spacing, centered on the macula. The macular scans were masked-exported by the external operator, randomized, and graded independently by one vitreoretinal surgeon (D.T.). For intraobserver reproducibility, the same examiner reanalyzed all SD-OCT imaging twelve weeks later, grading the scans only as pathological or healthy.

### Statistical analysis

For statistical analysis, data were analyzed and modeled by means of R language^14^. Descriptive data were expressed as the mean, standard deviation (SD) and percentage. Sensitivity (also called the true positive rate), specificity (also called the true negative rate), accuracy, false-positive and false negative rate were calculated. Furthermore, positive and negative predictive value, positive and negative likelihood ratio, and diagnostic odds ratio (DOR) were also determined. The diagnostic odds ratio is a measure of test performance and combines the strengths of sensitivity and specificity, as independent prevalence indicators, with the advantage of accuracy as a single indicator^15^. Although it was widely used in epidemiology to express the association between exposure and disease, it also can be applied to express the strength of association between test result and disease. DOR ranges from zero to infinity, although for useful tests it is greater than one, and higher diagnostic odds ratios are indicative of better discriminatory test performance^16^.

A non-parametric statistical analysis with Kendall’s Coefficient of Concordance (W) was calculated to analyze the measure of agreement among the different examiners^17^. It is a normalization of the statistic of the Friedman test and is used to determine the relationships between two sets of ranked data. The coefficient returns a value from 0 to 1, where 0 is no agreement, and 1 is a complete agreement. The correlation measure W has only a descriptive value, and, in this respect, it is important to verify the significance of the calculated value with an interferential test^17^. Therefore, a logistic regression model between the correlation index and the SD-OCT scans was assessed.

## Data Availability

The datasets generated during the current study are available from the corresponding author on reasonable request.
